# Glaucoma after Penetrating Keratoplasty: Incidence, Risk Factors, and Management

**DOI:** 10.1155/2011/951294

**Published:** 2011-11-30

**Authors:** Nilgun Yildirim, Huseyin Gursoy, Afsun Sahin, Ahmet Ozer, Ertugrul Colak

**Affiliations:** ^1^Department of Ophthalmology, Eskisehir Osmangazi University Medical Faculty, 26480 Eskisehir, Turkey; ^2^Department of Biostatistics, Eskisehir Osmangazi University Medical Faculty, 26480 Eskisehir, Turkey

## Abstract

*Purpose*. To report the incidence and risk factors for postkeratoplasty glaucoma (PKG), as well as its management. *Subjects and Methods*. 122 eyes, (43% with pseudophakic and aphakic bullous keratopathy (PABK)) which underwent penetrating keratoplasty (PK), were analyzed. *Results*. The rate of PKG development was 34% within 39 months of follow-up. PABK, corneal perforations, keratitis, and previous high intraocular pressure (PHIOP) were high risk factors for PKG. Glaucoma was controlled medically in 62% of PKG cases. Surgery (Ex-PRESS shunt in 63%) and diode laser cyclophotocoagulation were applied in others (38%). The rate of postoperative complications and graft survival was similar in eyes with and without PKG. *Conclusion*. PHIOP, preoperative diagnoses other than keratoconus, and corneal dystrophies were highly associated with PKG. Ex-PRESS shunts were effective in refractory PKG. If glaucoma is controlled, it is possible to obtain similar rates of graft survival and postoperative complications in eyes with and without PKG.

## 1. Introduction

There are many corneal transplantation techniques such as deep anterior lamellar keratoplasty and Descemet's stripping endothelial keratoplasty with many advantages over penetrating keratoplasty (PK), but PK is still the most common type of corneal transplant performed [1]. The leading indications for PK are keratoconus, bullous keratopathy (BK), corneal scars due to previous intraocular surgeries, infections, or trauma, corneal dystrophies, and graft failure [2]. In developing countries, corneal scars due to herpes simplex virus, presumed bacterial infections, or traumatic insults are more frequent indications for PK than the noninflammatory conditions such as keratoconus and corneal dystrophies [3]. The success of PK depends on many preoperative, intraoperative, and postoperative factors, including the health of the donor cornea, the indication for PK, suture techniques preferred, the quality of postoperative management, and the presence of high intraocular pressure (IOP) [1]. Postkeratoplasty glaucoma (PKG) is one of the challenging issues important for the survival of the graft. The incidence of PKG has been reported to range between 9% and 35% [4–9]. It has been reported to be one of the most serious complications following PK and the second leading cause of graft failure after graft rejection [10, 11]. Its diagnosis and management are much more difficult than the glaucoma cases with their own corneas [12]. 

The aims of our study were to report the incidence and risk factors for PKG and its management.

## 2. Materials and Methods

This was a retrospective study conducted at the Department of Ophthalmology, Eskisehir Osmangazi University Medical Faculty, Eskisehir, Turkey. The charts of 155 eyes that underwent PK between January 2007 and July 2010 were reviewed independently from the indication for PK. Out of these 155 eyes, 122 satisfied all the inclusion criteria. The inclusion criteria were follow-up period of at least 12 months after PK and well-documented IOP measurements at each visit. These 122 eyes were allocated to three groups depending on the indications for PK. Group 1 included 29 eyes of 24 patients (16 women and 8 men) with keratoconus or corneal dystrophies. Group 2 included 53 eyes of 51 patients (28 women and 23 men) with pseudophakic or aphakik BK. Group 3 included 40 eyes of 40 patients (20 women and 20 men) with indications other than those in groups 1 and 2. Indications for PK are represented in Table 1. 

PKG was defined as the persistence of raised IOP (>21 mmHg) or the requirement for increased treatment in patients with previous high intraocular pressure (PHIOP), one month after PK, in the presence of glaucomatous optic disc changes. All the procedures, namely, PK, glaucoma surgeries, and diode laser cyclophotocoagulation (DCPC), were performed by an experienced ophthalmologist (NY). 97 PK cases were performed under general anesthesia and the remaining under retrobulbar anesthesia. Standard surgical technique was used. The mean diameter of the donor corneal button was 8.0 mm (range, 7.5–8.5 mm), and the mean diameter of the recipient bed was 0.5 mm to 0.25 mm smaller than the donor corneal bed. Single continuous suture was preferred in most of the cases. In highly vascularized corneas, interrupted sutures were used. Additional procedures during PK were performed on an individual basis (Table 2). PK and cataract surgery combined with intraocular lens (IOL) implantation were performed in eleven eyes, and in two of these, PKG developed. Phacoemulsification (Phaco) and in-the-bag posterior chamber IOL (PCIOL) implantation were performed in four of these through the diseased cornea and open-sky extracapsular cataract extraction (ECCE) in the other seven. In the four cases that underwent ECCE surgery, PCIOL was implanted in the ciliary sulcus, and no IOL was implanted in the other three. Phaco and in-the-bag PCIOL implantation were performed in six eyes during the follow-up. Hydrophobic acrylic foldable IOL was inserted in the bag and poly(methyl methacrylate) (PMMA) IOL was preferred in the ciliary sulcus. IOL exchange or extraction was performed during PK in eight cases. Iris-claw lens (Ophtec) was implanted in these eight cases. 

Topical antibiotic eye drops four times/day for one month and topical prednisolone phosphate (0.5%) eye drops four times/day for up to one year, with gradually tapering doses, were routinely applied in all cases. Topical and/or systemic steroids in higher doses were applied if anterior segment inflammation and/or graft rejection occurred.

Topical beta-blockers, carbonic anhydrase inhibitors, and alpha-2 agonist were initiated in PKG cases. In PKG cases refractory to medical treatment, trabeculectomy, the Ex-PRESS shunt (with 50 micron lumen) implantation, the Ahmed glaucoma valve (AGV) implantation, or DCPC were performed under retrobulbar anesthesia. 5-Fluorouracil was applied intraoperatively in the trabeculectomy and Ex-PRESS shunt implantation. The Ex-PRESS shunts were implanted under partial-thickness scleral flap. AGV was implanted in the superotemporal quadrant beneath the sub-Tenons's space. The subconjunctival and sub-Tenons's portion of the tube was covered with a patch graft of donor dura matter. An informed consent was obtained from all subjects before surgery. The Tenets of the Declaration of Helsinki were followed, and the local medical ethics committee approved the study. 

All patients were followed up postoperatively with routine ophthalmic examinations at the first day, first week, first month, the third month, the sixth month, and every six months thereafter. The best-corrected visual acuities (BCVAs) in logMAR units and IOP pressure were assessed preoperatively and postoperatively at each visit. The BCVAs at the final visit were used for statistical and clinical analyses. For data analysis in the study, 2.2 logMAR, 2.3 logMAR, and 2.4 logMAR were used instead of hand movement (HM), perception of light (PL), and no perception of light (NPL), respectively. The IOP was measured using the Tono-Pen. The indications for PK, the presence of PHIOP, and the lens status were noted. The anterior segment examination was performed at each visit. The management modalities for PKG, the IOP before the initiation of glaucoma treatment, the IOP at the final visits, and the number of antiglaucomatous drugs applied were documented. Eyes were evaluated regarding the incidence and risk factors for developing PKG. 

The IOP before treatment was compared with IOP after treatment in medically and surgically treated cases separately using paired samples *t*-test. Odds ratios and 95% confidence intervals were calculated to determine the probability of developing PKG, using logistic regression analysis with PKG as the dependent variable, and the indications for PK, the lens status, and the PHIOP as independent variables. The incidence of graft rejection, graft failure, and post-keratoplasty infections in cases with PKG was compared with that without PKG using Yates' chi-square test. *P* value < 0.05 was required for statistical significance. Statistical analyses were performed using SPSS version 15.0 (SPSS Inc., Chicago, Ill, USA).

## 3. Results

122 eyes of 115 patients were reviewed. PKG developed in 42 (34%) of these eyes within 38.9 ± 14.3 (12–72) months of follow-up.

The mean preoperative and postoperative BCVA in eyes with PKG were 2.12 ± 0.25 and 1.6 ± 0.71 logMAR units, respectively (*P* = 0.001). The mean pre-operative and post-operative BCVA in eyes without PKG were 2.13 ± 0.16 and 1.17 ± 0.85 logMAR units, respectively (*P* = 0.001). The visual acuity was improved in 33/42 (79%) of the eyes with PKG and 64/80 (80%) of the eyes without PKG (*P* > 0.05). 

The number of eyes with PHIOP, post-keratoplasty glaucoma (PKG) and the PKG cases requiring medical or surgical treatment according to the indications of PK are represented in Table 3. Seventeen eyes (one in group 1, thirteen in group 2, and three in group 3) had a PHIOP. IOP was ≤21 mmHG with medication prior to PK in these seventeen eyes, but in thirteen of these PKG developed. In seven out of these thirteen, IOP was not controlled despite increased medication and glaucoma surgery was performed. 

The indications for PK other than keratoconus and corneal dystrophies, previous pseudophacia and aphakia, cataract surgery after PK, and PHIOP were highly associated with PKG (*P* < 0.05) (Table 4). Pseudophakia (including 36 posterior and ten anterior chamber IOL) prior to PK was present in 46 eyes and aphakia prior to PK in 26 eyes. 

Pre- and posttreatment IOP values in medically and surgically treated eyes are represented in Tables 5 and 6. IOP was >21 mmHG despite medical and surgical treatments in one case, in which trabeculectomy and DCPC were performed. 

The mean time interval between the diagnosis of PKG and PK was 12.8 ± 8.9 (2–36) months. Sixteen PKG cases were refractory to antiglaucomatous drugs. The AGV implantation was performed in three of these, the Ex-PRESS mini glaucoma shunt implantation in ten of these, and DCPC in two of these. DCPC was performed three months following trabeculectomy in one case (Table 6). 

Corneal graft rejection occurred in seven out of 42 PKG eyes and thirteen out of 80 eyes without PKG. Corneal graft failure developed in six out of these twenty cases in which rejection occurred. Fourteen cases responded to medical treatment. 

Corneal graft failure developed in twenty cases. Post-keratoplasty infections were responsible for failures in nine cases. The risk for developing corneal graft rejections, corneal graft failures, or infections following PK was similar in patients with and without PKG (Table 7). Regraft was performed in nine eyes.

## 4. Discussion

In the present study, the incidence of PKG was found to be 34% with 39 months of follow-up. Most of the PKG cases were diagnosed within a year following PK. Simmons et al. also reported an incidence of 34% of PKG following PK [9]. The mean time interval from PK to diagnosis of PKG was 24 weeks. The ten-year cumulative risk of PKG following PK was found to be 21% by Ing et al. [10]. The incidence of PKG was reported to be lower in the early post-operative period, but if long-term follow-up had be a possible, the rate probably would have increased [4, 5, 9]. 

The diagnosis of PKG is a challenging process due to difficulties in the measurement of IOP in the corneal graft and the possible occurrence of steroid-induced IOP elevations in the post-operative period [13, 14]. The Tono-Pen is the most accurate commercially available instrument for measurement of IOP in the early post-operative period, so the Tono-Pen was preferred in the present study [15]. The diagnosis of PKG was made if IOP rise persisted after one month following PK in the presence of glaucomatous optic disc changes. Temporary IOP elevations due to inflammatory processes can occur in the early post-operative period, and this can interfere with the diagnosis of PKG. In addition to this, the corneal edema, which is frequently observed in the early post-operative period, resolves after the first month, so that the IOP measurements are more accurate after one month from PK.

It has been reported that the incidence PKG is associated with the indications for PK [7]. Patients with pseudophakic BK, corneal perforation, and graft rejection were shown to be at high risk for PKG. Our findings were consistent with the previous studies [9, 16, 17]. In the present study, PKG developed in 20% of the patients with keratoconus or corneal dystrophies. There was only one PK case refractory to medical treatment in the keratoconus and corneal dystrophy group. The ratio was higher with other corneal pathologies such as pseudophakik BK, corneal perforations, and herpes keratitis. However, nine out of nineteen (47%) PK cases that developed in BK cases and six out of seventeen PK cases that developed in group 3 did not respond to antiglaucomatous drugs.

In the present study pseudophakia and aphakia prior to PK and combined surgery (phaco and IOL implantation) during the follow-up after PK were found to be the risk factors for PKG. The majority of the pseudophakic and aphakic cases were having BK, so it is not possible to consider pseudophakia and aphakia as independent risk factors. Inflammatory processes associated with the surgery, the IOL material, the peripheral anterior synechia formation, and the effects of aphakia and pseudophakia on the angle structures are the most probable explanations for the increased PK incidence in these cases [8, 9, 18].

In thirteen (76%) of the cases with PHIOP, PKG developed. In seven of these cases, glaucoma surgery was performed to lower the IOP, whereas antiglaucomatous drugs were effective in the other six. The finding was consistent with the previous studies in which PHIOP was shown to be a major risk factor for PKG [19]. 

Sixteen PKG cases (38%) were refractory to medical treatment, and trabeculectomy AGV implantation, Ex-PRESS mini glaucoma shunt implantation, and DCPC were performed in these. In all of the cases, except one, IOP was controlled with the surgical interventions. The AGV implantation was shown to be associated with graft failure due to tube-corneal endothelium touch and the instability of the tube in several studies [20]. The Ex-PRESS mini glaucoma shunt implantation was the most preferred surgical procedure (62%) in our study. It was successfully implanted in ten cases lowering mean IOP from 29 to 13 mmHg. It is a small nonvalved device that is very stable in the anterior chamber [21]. The efficacy of the device has been reported to be similar with trabeculectomy in healthy corneas [21]. Ates et al. achieved success rate of 93% with the Ex-PRESS shunt in PKG cases [22]. The Ex-PRESS shunt may be an alternative treatment in PKG cases resistant to medical treatment. It has many advantages over trabeculectomy and conventional glaucoma drainage devices. First, it is a simple and less invasive procedure compared to trabeculectomy. Second the risk of intraoperative and postoperative inflammation and complications is low [21]. Finally the risk of endothelial cell loss associated with AGV implant is negligible with the Ex-PRESS shunt [21]. 

Inadequate control of IOP after PK is an important cause of graft failure [23]. In the present study, the IOP of ≤21 mmHg was obtained by medical treatment, surgical treatment, or DCPC in the majority of the PKG cases. The rates of post-operative complications including graft rejection episodes, graft failures, and post-keratoplasty infections were similar in patients with and without PKG. The success rate of visual acuity improvement was the same in patients with and without PKG.

There are some limitations of the current study. The number of eyes that underwent PK due to non-inflammatory conditions (keratoconus and corneal dystrophies) was much less than the eyes that underwent PK due to other pathologies. Pseudophakik and aphakik BK comprised 43% of the study group, whereas the noninflammatory conditions comprised 24% of the study group. There are several explanations for the lower percentage of the non-inflammatory conditions in the present study. First, this retrospective study was conducted at our ophthalmology department, which is considered to be one of the national referral centers for corneal diseases, so many complicated cases including BK and corneal perforations have been referred to our clinic. Second, the patient satisfaction after PK in the eyes with the non-inflammatory conditions is usually higher than that after PK in eyes with inflammatory conditions, such as BK, keratitis, and corneal perforations. Therefore, there are many patients who fail to attend *their appointment* after a successful PK, and this affected the distribution of the pre-operative diagnoses in the present study. The distribution of the pre-operative diagnosis caused overestimation of the incidence of PKG, since BK was one of the high risk factors for the development of PKG. The graft failure was also overestimated. In a study including 3640 eyes that underwent PK for the first time, the survival of grafts was reported to be 90% at five years. The highest survival rate was documented in grafts for eyes with non-inflammatory conditions and the lowest in grafts for eyes with BK, being 70% at five years [24]. The small number of eyes with non-inflammatory conditions in the current study was the possible explanation for the high incidence of graft failure after a mean follow-up of 39 months. The incidence of post-keratoplasty infections was 10% in the present study. It was reported to range from 2% to 12% of eyes undergoing PK [25–27]. The large percentage of eyes undergoing PK due to inflammatory conditions (76%) may explain the high incidence of post-keratoplasty infections. 

In conclusion, PKG developed in one out of three patients who underwent PK. PHIOP, pseudophakik BK, pseudophakia, aphakia, corneal perforations, and corneal scars were highly associated with PKG, whereas PKG was less likely to develop in cases with keratoconus and corneal dystrophies. In PKG cases refractory to medical treatment, variable glaucoma surgeries and DCPC may be applied. The Ex-PRESS shunt implantation may be the first-choice surgical procedure for refractory PKG. If IOP is adequately controlled in PKG, it may be possible to obtain similar rates of graft survival and post-operative complications in eyes with and without PKG.

## Figures and Tables

**Table 1:** Indications for penetrating keratoplasty.

	Indications	Number of eyes	Percentage
Group 1	Keratoconus	17	14%
	Corneal dystrophies	12	10%

Group 2	Pseudophakic/aphakic bullous keratopathy	53	43%

Group 3	Herpes simplex keratitis	7	6%
	Corneal scars due to corneal perforation	9	7%
	Corneal scars due to presumed infections	13	10.5%
	Corneal graft failure	6	5%
	Silicon keratopathy	3	2.5%
	Spontaneous corneal perforation	2	2%

**Table 2:** The number of eyes that underwent additional procedures during penetrating keratoplasty.

	Group 1	Group 2	Group 3
Anterior vitrectomy	0	3	2
Synechiolysis	0	4	6
Pupilloplasty	0	1	2
Cataract surgery	2	0	9
Intraocular lens exchange	0	3	1
Intraocular lens extraction	0	3	1

**Table 3:** The number of eyes (%) with previous high intraocular pressure (PHIOP) and post-keratoplasty glaucoma (PKG) and the PKG cases requiring medical or surgical treatment according to the indications of penetrating keratoplasty.

	Group 1	Group 2	Group 3
Follow-up time (months)	39.0 ± 10.9	42.7 ± 13.2	33.6 ± 13.9
Age (years)	44.8 ± 16.9	66.8 ± 11.9	56.5 ± 19.6
PHIOP (%)	1/29 (3%)	13/53 (25%)	3/40 (8%)
Post-keratoplasty glaucoma (%)	6/29 (20%)	19/53 (36%)	17/40 (42%)
Medically treated PKG cases	5	10	11
Surgically treated PKG cases	1	8	5
Diode-laser-applied cases	0	2	1

**Table 4:** Odds ratio and 95% confidence interval from binomial regression of the likelihood of developing post-keratoplasty glaucoma (PKG) (versus without PKG) on indications for penetrating keratoplasty (PK), the lens status, and previous high intraocular pressure (PHIOP).

		Univariate binomial logistic regression analysis	
	Variables	Odds ratio	95% confidence interval
Indications for PK	Group 1	1	
	Group 2	2.1	0.7–6.2
	Group 3	2.8	0.9–8.4

Lens status	Phakic	1	
	Aphakia before PK	2.1	0.7–6.6
	Pseudophakia before PK	2.6	0.9–7.3
	Combined cataract surgery	1.3	0.3–6.4
	Cataract surgery after PK	2.4	0.4–17.2

PHIOP	Nonexisting	1	
	Existing	8.5	2.6–28.3

**Table 5:** The mean pre- and posttreatment intraocular pressures (IOPs) in mmHg.

	Number of cases	Pretreatment IOP	Posttreatment IOP	*P* value
Medically treated cases	26	26.9 ± 3.0	15.9 ± 1.8	0.001

Surgically treated cases including diode laser applications	16	29.5 ± 4.7	14.2 ± 4.1	0.001

**Table 6:** The management of refractory post-keratoplasty glaucoma cases. The mean intraocular pressures (IOPs) in mmHg, the mean number of antiglaucomatous drugs before and after treatment.

	Number of cases	IOP before	IOP after	Drugs Number of before	Drugs Number of after
Trabeculectomy	1	36	18	3	2
Ex-PRESS shunt	10	28.9 ± 5.3	12.9 ± 3.1	2.6 ± 0.8	0.8 ± 1.1
AGV implant	3	29	15	3	2
Diode laser	3	32	17	3	2

AGV: Ahmed glaucoma valve.

**Table 7:** Incidence of graft rejection, graft failure, and post-keratoplasty infections in cases with post-keratoplasty glaucoma (PKG) versus in cases without PKG.

	With PKG	Without PKG	*P* value
Graft rejection	7/42 (17%)	13/80 (16%)	0.9
Graft failure	7/42 (17%)	13/80 (16%)	0.9
Post-keratoplasty infections	4/42 (10%)	8/80 (10%)	0.9
